# Optimization of immunolocalization of cell cycle proteins in human corneal endothelial cells

**Published:** 2011-12-29

**Authors:** Zhiguo He, Nelly Campolmi, Binh-Minh Ha Thi, Jean-Marc Dumollard, Michel Peoc’h, Olivier Garraud, Simone Piselli, Philippe Gain, Gilles Thuret

**Affiliations:** 1 “Biology, Engineering and Imaging of Corneal Grafts” Laboratory EA2521, SFR143, Faculty of Medicine, University Jean Monnet, Saint-Etienne, France; 2 Eye Bank of Saint-Etienne, Auvergne Loire Blood Center, Saint-Etienne, France; 3 Department of Pathology, University Hospital of Saint-Etienne, Saint-Etienne, France

## Abstract

**Purpose:**

En face observation of corneal endothelial cells (ECs) using flat-mounted whole corneas is theoretically much more informative than observation of cross-sections that show only a few cells. Nevertheless, it is not widespread for immunolocalization (IL) of proteins, probably because the endothelium, a superficial monolayer, behaves neither like a tissue in immunohistochemistry (IHC) nor like a cell culture in immunocytochemistry (ICC). In our study we optimized IL for ECs of flat-mounted human corneas to study the expression of cell cycle-related proteins.

**Methods:**

We systematically screened 15 fixation and five antigen retrieval (AR) methods on 118 human fresh or stored corneas (organ culture at 31 °C), followed by conventional immunofluorescence labeling. First, in an attempt to define a universal protocol, we selected combinations able to correctly localize four proteins that are perfectly defined in ECs (zonula occludens-1 [ZO-1] and actin) or ubiquitous (heterogeneous nuclear ribonucleoprotein L [hnRNP L] and histone H3). Second, we screened protocols adapted to the revelation of 9 cell cycle proteins: Ki67, proliferating cell nuclear antigen (PCNA), minichromosome maintenance protein 2 (MCM2), cyclin D1, cyclin E, cyclin A, p16^Ink4a^, p21^Cip1^ and p27^Kip1^. Primary antibody controls (positive controls) were performed on both epithelial cells of the same, simultaneously-stained whole corneas, and by ICC on human ECs in in vitro non-confluent cultures. Both controls are known to contain proliferating cells. IL efficiency was evaluated by two observers in a masked fashion. Correct localization at optical microscopy level in ECs was define as clear labeling with no background, homogeneous staining, agreement with previous works on ECs and/or protein functions, as well as a meaningful IL in proliferating cells of both controls.

**Results:**

The common fixation with 4% formaldehyde (gold standard for IHC) failed to reveal 12 of the 13 proteins. In contrast, they were all revealed using either 0.5% formaldehyde at room temperature (RT) during 30 min alone or followed by AR with sodium dodecyl sulfate or trypsin, or pure methanol for 30 min at RT. Individual optimization was nevertheless often required to optimize the labeling. Ki67 was absent in both fresh and stored corneas, whereas PCNA was found in the nucleus, and MCM2 in the cytoplasm, of all ECs. Cyclin D1 was found in the cytoplasm in a paranuclear pattern much more visible after corneal storage. Cyclin E and cyclin A were respectively nuclear and cytoplasmic, unmodified by storage. P21 was not found in ECs with three different antibodies. P16 and p27 were exclusively nuclear, unmodified by storage.

**Conclusions:**

IL in ECs of flat-mounted whole human corneas requires a specific sample preparation, especially to avoid overfixation with aldehydes that probably easily masks epitopes. En face observation allows easy analysis of labeling pattern within the endothelial layer and clear subcellular localization, neither of which had previously been described for PCNA, MCM2, or cyclin D1.

## Introduction

**Figure 1:**
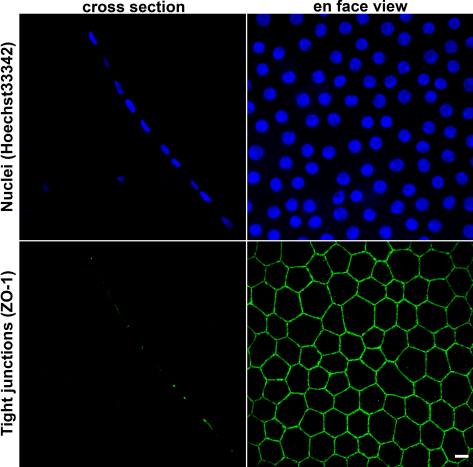
Hoechst 33342 nuclei staining and tight-junction immunostaining with zonula occludens-1 (ZO-1) antibody illustrate the differences between conventional immunohistochemistry on a corneal cross-section where only a few endothelial cells are partially visible, and en face view showing a wide field of intact cells. Original magnification 40×. Bar 10 μm.

The corneal endothelium functions as a permeability barrier that restricts the movement of water and solutes into the hydrophilic stroma to control corneal transparency [1]. It is composed of a monolayer of flat (approximately 5 μm) tessellated joint cells forming a mosaic of mainly hexagonal elements (Figure 1). In the adult human, the proliferative capacities of endothelial cells (ECs) are practically nil and cannot offset cell losses in physiologic or pathological cases. Indeed, no increase in EC number has been clinically documented. Although in physiologic conditions the number of ECs decreases very slowly, by about 0.6% per year during adulthood [2,3], this pace accelerates dramatically in several corneal diseases (mainly Fuchs’ dystrophy, the most frequent endothelial primary dystrophy) and after accidental or surgical traumatisms: endothelial cell density (ECD) falls below a threshold of 300 to 500 cells/mm^2^ (depending on the kinetics of loss) and an irreversible corneal edema occurs, causing permanent visual loss. During corneal storage by eye banks, be it in short-term cold storage at 4 °C in Optisol or in long-term storage at 31 °C to 34 °C in organ culture (OC) media, EC loss also accelerates strongly, causing a decrease in graft quality. In the past two decades Joyce and colleagues have highlighted, mainly based on proteomic studies, that adult ECs in vivo are arrested in the G_1_ phase of the cell cycle (reviewed in [4]). However in parallel, they also demonstrated, with other, that ECs retain proliferative capacity [5] even in the elderly, but with an age-related decrease [6,7]. Primary EC cultures from adult donors of all ages can also be routinely obtained in vitro [8].

Stimulation and control of EC proliferation would have high therapeutic impact, making it possible to increase ECD directly in vivo in patients with low ECD and to improve the quantity and quality of donor corneas by increasing ECD ex vivo during storage in eye banks, or by facilitating large-scale development of bioengineered endothelial grafts. Identification of the proteins involved in cell cycle arrest is key to these future EC therapies.

Immunolocalization (IL) of proteins has been previously used, as a basic tool, on corneal endothelium. Generally, ECs can be observed either on corneal cross-sections or on flat-mounts, as the EC monolayer of 300,000 to 400,000 tessellated cells on the innermost surface of the cornea is easily visible by en face observation. Although standard immunohistochemistry (IHC) on corneal cross-sections is the prevalent method in the literature, it allows visualization of only a few cells, not a complete view of the unmodified endothelium (Figure 1). Moreover, it does not always give a clear subcellular localization of proteins of interest, a condition required for a proper interpretation [9]. On the contrary, IL on flat-mounted corneas theoretically appears far more informative. It gives access to the whole intact tissue with a possibility to detect regional differences in expression patterns (for example a centripetal gradient) as well as to detect rare isolated cells among an apparently homogeneous cell population. Nevertheless, to date it has been used in very few studies and for few proteins. Moreover, in the only paper reporting the use of both cross-sections and flat-mounts with the same antibodies, immunostaining was not identical, with generally fewer positive results on flat-mounts [10].

In the present study, we systematically screened immunostaining protocols to select those able to correctly reveal proteins in ECs of intact flat-mounted corneas. Fixation and antigen retrieval (AR) are two fundamental steps in all immunostaining processes. They strongly influence the ability of specific antibodies to recognize their targets (retention of antigenicity) while offering good preservation of ultrastructure. Cross-linking fixatives (aldehydes) act by creating covalent chemical bonds between proteins, mainly between lysine residues [11]. They also anchor soluble proteins to the cytoskeleton. Formaldehyde is the most common fixative in histology. Four percent formaldehyde (equivalent to the neutral-buffered formalin (10%) used for clinical pathology) appears as a gold standard in most studies, including those on corneal endothelium. Precipitating, also called denaturing, fixatives (ethanol, methanol, and acetone) reduce the solubility of proteins and disrupt the hydrophobic interactions involved in the tertiary structure of proteins. They are commonly used for immunocytochemistry (ICC), alone or in combination. Metallic fixatives, which denature the proteins and create weak bonds between them, are rarely employed alone in ICC or IHC. Nevertheless, HgCl_2_ or ZnCl_2_ containing formaldehyde solutions improve nuclear morphology with various effects on antigenicity [12]. AR, either by heating in specific buffers, enzymatic digestion or chemical reagents, helps unmask certain hidden epitopes. It can be helpful or even crucial during IHC of formalin-fixed paraffin-embedded tissues [13]. Heat-induced antigen retrieval (HIAR), known to hydrolyse protein cross-linking that occurred during aldehyde fixation (but not only [14]), is the most common AR technique [15], but enzymatic digestion by trypsin that cleaves peptide chains mainly at the carboxyl side of lysine, and protein denaturants like sodium dodecyl sulfate (SDS) and urea, have also been used.

In this study, we present optimization of fixation and AR methods for ECs of flat-mounted whole human corneas, and results obtained on a very large series of corneas for IL of proteins involved in cell cycle control.

## Methods

### Human corneas

Corneas were retrieved by in situ excision, the routine method for clinical use. Fifty corneas were obtained from the Department of Anatomy of the Faculty of Medicine of Saint-Etienne (body donation to science). Bodies were refrigerated at 4 °C in conditions comparable with those used for donation for clinical use. Donor age (mean±standard deviation, range, median) was 81±11 years, (46 to 99, 82), and time from death to procurement was 27±14 h (7 to 48, 23). Corneas were fixed immediately after procurement and further processed (group 1 of fresh corneas). Seventy-six other corneas were obtained from the Eye Bank of Saint-Etienne, Saint-Etienne, France. Corneas were not suitable for corneal graft because of inconclusive donor serologies, but had a normal endothelium. Corneas were stored in OC at 31 °C for 25±9 days (7 to 35, 27) in CorneaMax medium (Eurobio, Les Ulis, France) before fixation and processing for immunostainings (group 2 of stored corneas). Donor age was 73±12 years (42 to 94, 76), and death-to-procurement time always less than 24 h (13±6 h [4 to 24, 12]). Handling of the donor tissues adhered to the tenets of the Declaration of Helsinki of 1975 and its 1983 revision in protecting donor confidentiality.

### Primary endothelial cell cultures

Using eight OC corneas from the group described above, ECs were isolated and cultured according to protocols published elsewhere [8]. Briefly, Descemet’s membrane with the attached ECs was retrieved under a dissecting microscope, and incubated in 0.2 mg/ml ethylenediaminetetraacetic acid (EDTA) in Hanks’ balanced salt solution (Eurobio), pH 7.4, for 1 h at 37 °C. After centrifugation, cells were resuspended in OPTIMEM-1 (Invitrogen Life technologies, St-Aubin, France) supplemented with 8% fetal bovine serum (Eurobio), 40 ng/ml of fibroblast growth factor, 5 ng/ml of epidermal growth factor, 20 ng/ml of nerve growth factor (the three from Invitrogen), 20 mg/ml of ascorbic acid, 0.005% human lipids, 200 μg/l of calcium chloride, 0.08% chondroitin sulfate, 1% RPMI-1640 multiple vitamin solution, and 50 μg/ml gentamicin (the last six from Sigma, St Quentin Fallavier, France). Cells were incubated in six-well tissue culture plates at 37 °C in a 5% carbon dioxide humidified atmosphere. Medium was changed every other day. After primary cultures reached confluence, cells were transferred to a non-precoated T25 flask and subcultured until confluence. Then, 2000 cells per well were seeded in non-precoated 96-well tissue culture plates and cultured until subconfluence before being used for ICC. These cultures were used as controls (see below).

### Study design

The work comprised two steps. First, we studied whether a universal corneal preparation existed for IL in ECs of flat-mounted corneas. We therefore chose four proteins known to be expressed by ECs independently of their proliferative status and localized in four different cell compartments. They were either perfectly characterized in human ECs or ubiquitous proteins: (1) zonula occludens-1 (ZO-1) is a peripheral membrane protein associated with tight junctions [16]; (2) actin is one of the most conserved cytoplasmic proteins in eukaryotes and is distributed in linear circumferential strands that form a hexagonal belt beneath plasmic membrane in ECs [17]; (3) heterogeneous nuclear ribonucleoprotein L (hnRNP L) is present in the nucleoplasm as part of the hnRNP complexes that play a major role in the formation, packaging, processing, and function of mRNA; (4) bound with DNA, histone H3 is one of the basic nuclear proteins responsible for the nucleosome structure of chromosomal fibers in eukaryotes.

Considering the large number of fixatives and AR methods to assess, a systematic experiment matrix was built to minimize the number of human corneas necessary. Fixatives and AR were chosen from the general histology and cytology literature [18,19] as well as from current laboratory practices. They were classified as shown in Table 1. To increase the number of experiments while minimizing the number of human corneas necessary, each cornea was cut into eight pie-shaped wedges before or after fixation. Fixatives were then successively assessed and preselected once correct localization seemed to be achieved. Also to reduce the number of corneas necessary, secondary controls and labeling controls (see below) were done only once a protocol had been preselected. When labeling of ECs and positives controls seemed specific in terms of localization but faint and/or non-homogeneous (not all cells), AR methods were assessed successively with the preselected fixative. In case of complete negativity for all fixation protocols, AR methods were assessed only with 0.5% formaldehyde fixation (which, during the previous experiments in the study, had proved to be the most efficient fixative overall).

**Table 1:** Fixation protocols and antigen retrieval methods assessed on whole corneas.

**Number**	**Fixation protocols**
1	4% formaldehyde*, in PBS, pH 7.45 30 min at 4 °C
2	0.5% formaldehyde*, in PBS, pH 7.45 30 min at 4 °C
3	4% formaldehyde*, in PBS, pH 7.45 30 min at RT
4	0.5% formaldehyde*, in PBS, pH 7.45 30 min at RT
5	100% methanol 30 min at −20 °C
6	100% methanol 30 min at RT
7	100% acetone 30 min at −20 °C
8	100% acetone/100% methanol (1:1 v/v) 30 min at −20 °C
9	100% acetone/100% methanol (1:1 v/v) 30 min at RT
10	Carnoy I** 30 min at −20 °C
11	Carnoy I** 30 min at RT
12	Bouin modified*** 45 min at RT
13	0.5% Zn(OAc)_2_ + 0.5% ZnCl_2_ + 0.05% Ca(OAc)_2_ in 0.1M Tris base buffer 45 min at RT (Tris-Zinc)
14	HgCl_2_ 6 g in formalin (37%–40%) 10 ml + distilled water 90 ml 45 min at RT
15	ZnCl_2_ 0.16 g + PFA 4 g + NaCl 5 g in distilled water 100 ml 45 min at RT
**Number**	**Antigen retrieval methods**
1	0.1 M citrate buffer pH 6, 4 h at 55 °C§
2	0.01 M EDTA buffer pH 10, 4 h at 55 °C§
3	0.1% trypsin in PBS, 5 min at 37 °C
4	4 M urea in water, 4 h at RT
5	0.5% sodium dodecyl sulfate in water, 5 min at RT

PBS phosphate buffer saline; RT room temperature; * always made fresh from paraformaldehyde; **100% ethanol/100% acetic acid (3:1 v/v); ***75 ml picric acid saturated in water + 25 ml 4% PFA+5 ml acetic acid; ^§^ the highest temperature that did not induce shrinking of fixed corneas.

Second, we applied the previously optimized protocols to 9 proteins involved in cell cycle: three proteins known as proliferation markers (Ki67, proliferating cell nuclear antigen [PCNA], and mini chromosome maintenance protein 2 [MCM2]); five proteins involved in controlling cell cycle phase G_1_ (cyclin D1, cyclin E, p16^INK4a^ (p16), p21^WAF1/Cip1^ (p21), and p27^Kip1^ (p27)); and cyclin A for the S phase. All had previously been studied in human corneas, but only Ki67 and MCM2 on flat-mounted whole corneas. Protocols selected after step one were applied to each of the 9 target proteins. If staining proved unsatisfactory, the systematic selection process was applied from the beginning as in step one, excluding the fixatives and AR methods that consistenly had negative results during step one.

### Immunolocalization on flat-mounted whole corneas

Corneas were rinsed in PBS before fixation. All tissues were fixed immediately after retrieval or after being removed from OC medium, as a delay between retrieval and fixation is detrimental for immunostaining results [11]. To increase the number of experiments, each cornea was cut into eight pie-shaped wedges before or after fixation, but we occasionally verified that the same results were observed for entire corneas (data not shown) to eliminate artifacts caused by the cut and border effects. Cell membranes were permeabilized by 1% Triton x-100 in PBS for 5 min at room temperature (RT) after fixation. This step was omitted when methanol, acetone or SDS was used, as they alter lipids by themselves. AR methods were then applied each time necessary (for details, see below). The non-specific binding sites were then saturated by incubation with goat serum for 30 min at 37 °C. Primary and secondary antibodies were diluted in PBS containing 2% bovine serum albumin (BSA) and 2% goat serum. Primary antibodies were incubated for 1 h at 37 °C. Corneal pieces were totally immersed in 100 µl of this solution in eppendorf microtubes to expose both endothelial and epithelial surfaces. Incubation with secondary antibodies diluted 1:500 in PBS containing 2% BSA and 2% goat serum was done for 45 min at 37 °C. Nuclei were finally counterstained with Hoechst 33342 (Sigma) 10 µg/ml in PBS at RT for 2 min. Three rinses in PBS were performed between all steps, except between saturation of non-specific protein binding sites and incubation with primary antibody. The corneal piece was finally placed on a glass slide, covered with PBS and gently flattened using a large glass coverslip retained by adhesive tape. Experiments were done at RT unless otherwise stated. Images were captured by a fluorescence inverted microscope IX81 (Olympus, Tokyo, Japan) equipped with the Cell^P imaging software (Soft Imaging System GmbH, Munster, Germany). This non-confocal microscope was chosen to obtain large-field images able to tolerate the residual tridimensional (3D) aspect of cell layers after flat-mounting.

The primary antibodies and their dilutions are listed in Table 2. Whenever possible, they were chosen from among antibodies validated for ICC by their manufacturers, which seemed closest to our technique. Non-specific rabbit and/or mouse IgG (ref 02–6102 and 02–6502, respectively; Zymed, Carlsbad, CA,) were used as primary antibodies for negative controls (i.e., secondary antibody control [20]). Complementarily, labeling control was performed by omitting primary and secondary antibodies, to identify potential autofluorescence. Secondary antibodies were Alexa Fluor 488 goat anti-mouse IgG or/and Alexa Fluor 555 goat anti-rabbit IgG (Invitrogen, Eugene, OR).

**Table 2:** List of primary antibodies.

**Target proteins**	**Animal source**	**Clonality**	**Laboratory**	**Reference**	**Dilution**
ZO-1	mouse	monoclonal	Zymed, Carlsbad, CA	33–9100	1/200
actin	rabbit	polyclonal	Sigma, Saint Louis, MO	A2066	1/200
hnRNP L	mouse	monoclonal	Abcam, Cambridge, UK	ab6160	1/200
histone H3	rabbit	polyclonal	Abcam	ab1791	1/200
Ki67 clone MIB-1	mouse	monoclonal	Dako, Glostrup, Denmark	M7240	1/100
PCNA	rabbit	polyclonal	Abcam	ab18197	1/200
MCM-2	rabbit	polyclonal	Abcam	ab31159	1/200
*cyclin D1	rabbit	monoclonal	Abcam	ab16663	1/200
*cyclin D1	rabbit	polyclonal	Abcam	ab31450	1/200
cyclin E	rabbit	polyclonal	Abcam	ab7959	1/100
cyclin A	mouse	monoclonal	Abcam	Ab38	1/200
p16^INK4a^	rabbit	polyclonal	Interchim, Fremont, CA	#RB-025-PABX	1/200
*p21^Cip/WAF1^	mouse	monoclonal	Cell Signaling, Danvers, MA	#2946	1/200
*p21^Cip/WAF1^	rabbit	polyclonal	Santa Cruz, Santa Cruz, CA	sc-397	1/200
*p21^Cip/WAF1^	mouse	monoclonal	Zymed, San Francisco, CA	33–7000	1/200
p27^kip1^	mouse	monoclonal	Sigma	P2092	1/200

*For cyclin D1 and p21, different antibodies were tested because of initial negative results.

### Controls for primary antibody specificity

To perform the equivalent of a primary antibody control [20] as an indicator for antibody specificity, immunostaining results in ECs of flat-mounted corneas were compared with those of two types of cells: epithelial cells of the same flat-mounted corneal piece, and primary cultures of ECs studied with ICC. Epithelial cells, known to form a highly proliferative layer during OC [21], served as positive controls for the whole process with predictable patterns of expression for proliferation markers and proteins involved in cell cycle control. Primary EC cultures served as positive controls of the same cell type, using conventional ICC techniques.

### Immunocytochemistry on endothelial cell cultures

Nonconfluent cell cultures were rinsed in PBS and fixed with 4% formaldehyde in PBS at RT for 15 min without any subsequent AR, except for cyclin A which required the AR with 0,5% SDS in water for 5 min at RT. Permeabilization, saturation of the non-specific binding sites, immunoreactions, and counterstaining were performed as described above for flat-mounts.

### Efficiency criterion

Protocols were deemed optimized once labeling exhibited correct localization at light microscopic level, with homogeneous staining, without zone effect (the first 100 μm near the cutting edges were excluded), and absence of staining in secondary antibody and labeling controls. Defining correct localization was easy for ZO-1, Actin, hnRNP L, and histone H3. For the 9 cell cycle-related proteins, for which no absolute certainty existed, localization was considered correct when it agreed with their fundamental characteristics and by comparison with the staining patterns in epithelial cells and EC cultures (both primary antibody controls). Homogeneous staining was the last factor required. In case of equivalence between protocols in terms of specificity, the brightest staining was selected. Whatever the result (positive or negative), each combination was assessed on three independent corneas. The experiment was done twice more before a protocol was rated “optimized.” Immunostainings were evaluated independently by two observers experienced in immunohistochemical assessment (Z.H., G.T.) on digital pictures blind to the preparation used. Subcellular localization, extent, homogeneity and intensity (signal/noise ratio) of immunostainings were considered. Intensity of staining was judged weak, moderate, or strong. In case of disagreement, a third judge (P.G.) reviewed the pictures.

## Results

### Optimization of immulocalization

#### ZO-1, actin, hnRNPL, and histone H3

**Figure 2:**
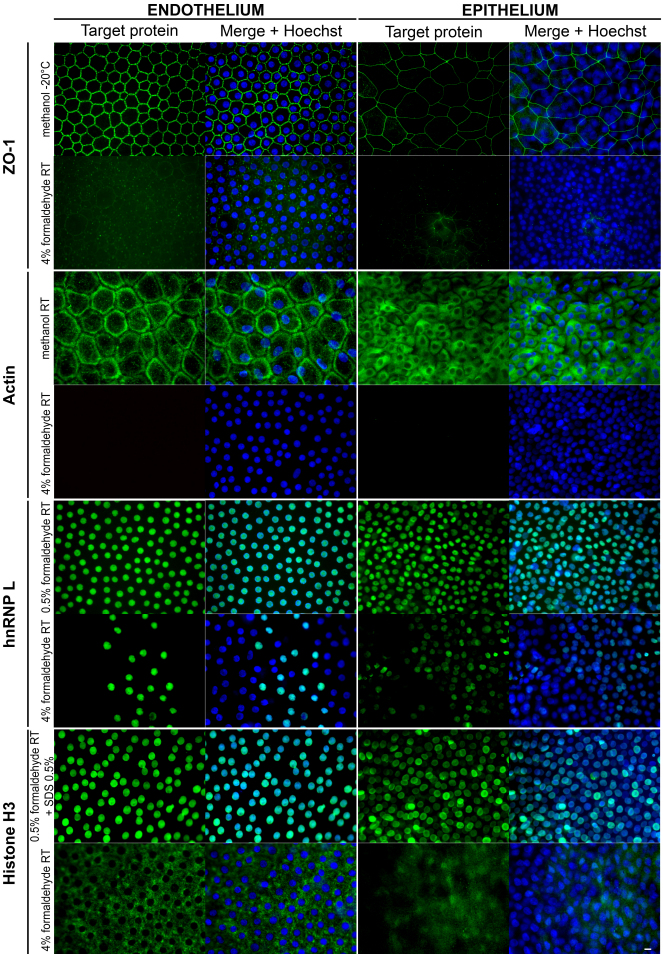
Optimization of immunolocalization of ZO-1, actin, hnRNP L, and histone H3 in endothelial cells on flat-mounted whole human corneas. Only the best combination is shown in the first line, compared with the 4% formaldehyde usually considered as the gold standard in immunohisto- and cytochemistry. Original magnification 40×. Bar 10 μm.

Standard IHC fixation with 4% formaldehyde (at 4 °C or RT) gave unacceptable results for the four proteins: absence of staining for actin, very weak staining or heterogeneous pattern for hnRNPL and ZO-1, or aberrant pattern (cytoplasmic instead of nuclear) for H3. These results clearly demonstrated the need for optimization. Using systematic testing, we found several protocols that provided the expected localization for the four proteins in ECs and epithelial cells, at least for the most superficial layers (Figure 2 and Table 3). Results were similar for both fresh and stored corneas (data not shown). Moreover, results of each protocol, whether correct or not, were always coherent between endothelium and epithelium: no discrepancy (correct localization on one side with aberrant staining on the other) was ever observed, confirming that epithelium can serve as a primary antibody control. Negative controls (with irrelevant antibodies and without antibodies) remained constantly unstained. Fixation in pure methanol at RT for 30 min appeared the best compromise to localize the four proteins present in four different cell compartments, even if not optimal for both nuclear proteins. AR greatly improved H3 labeling.

**Table 3:** Optimization of immunolocalization of ZO-1, actin, hnRNP L, and histone H3 in endothelial cells on flat-mounted whole human corneas.

**Target protein**	**Classification**	**Selected protocols**
ZO-1	best	methanol at −20 °C
** **	second choice	methanol at RT; 0.5% formaldehyde at RT; Carnoy I at −20 °C
Actin	best	methanol at RT
** **	second choice	Carnoy I at −20 °C
hnRNP L	best	0.5% formaldehyde at RT
** **	second choice	methanol at RT
histone H3	best	0.5% formaldehyde + AR with 0.5% SDS in water 5 min at RT *
** **	second choice	methanol at RT

* SDS: Sodium dodecyl sulfate. In these cases fixation was prolonged during 1 h to avoid endothelial cell detachment induced by SDS with short time fixation. For all other cases, fixation during 30 min.

#### Cell cycle-related proteins

Methanol for 30 min at RT, found to be the best compromise in the previous step, was not able to provide correct IL for the 9 proteins of interest, indicating that antibody/antigen specific preparations were probably necessary. Selected protocols are summarized in Table 4. There was no ubiquitous protocol but 0.5% formaldehyde alone was nevertheless usable for eight out of nine cases, even if it was not optimal for four antibodies. Notably, 0.5% formaldehyde alone was the only solution for p21 and p27 antibodies. AR strongly improved immunostaining quality (brightness and contrast) for PCNA, cyclin E, and p16. It was even the only efficient solution for cyclin A. Each time AR was necessary, it increased the risk of EC detachment from Descemet membrane, so fixation time was increased to 1 h to reduce this risk. As for ZO-1, Actin, hnRNPL, and H3, results of all assessed combinations were coherent between endothelial and epithelial cells of the same cornea. As we could not obtain cyclin D1 or p21 staining in ECs of flat-mounts with any of the protocols with the antibodies initially chosen, we assessed other antibodies using the same procedure. Staining of positive controls was always comparable between antibodies of the same target, using the same protocols.

**Table 4:** Optimization of immunolocalization for cell cycle-related proteins in endothelial cells on flat- mounted whole human corneas.

**Target protein**	**Classification**	**Selected protocols**
Ki67	best	0.5% formaldehyde
** **	alternatives	4% formaldehyde; methanol
PCNA	best	0.5% formaldehyde 60 min at RT + AR with 0.5% SDS 5 min at RT
** **	alternatives	0.5% formaldehyde; methanol
MCM-2	best	0.5% formaldehyde
** **	alternatives	/
cyclin D1 (abcam 31450)	best	Carnoy I at −20 °C
** **	alternatives	0.5% formaldehyde
cyclin E	best	0.5% formaldehyde 60 min at RT + AR with 0.1% trypsin 5 min at 37 °C
** **	alternatives	0.5% formaldehyde
cyclin A	best	0.5% formaldehyde 60 min at RT + AR with 0.5% SDS 5 min at RT
** **	alternatives	/
P16	best	0.5% formaldehyde for 60 min at RT + AR with 0.5% SDS 5 min at RT
** **	alternatives	0.5% formaldehyde
P21 (cell signaling #2946)	best	0.5% formaldehyde
** **	alternatives	/
P27	best	0.5% formaldehyde
** **	alternatives	/

Fixation was at RT during 30 min, unless otherwise stated. /=absence of second choice protocol.

For ICC on primary EC cultures, 4% formaldehyde gave reproducible correct IL for all proteins tested except for cyclin A, which required additional AR with 0.5% SDS in water for 5 min at RT.

#### Summary of optimized protocols obtained on flat-mounted whole corneas

Fixation with 0.5% formaldehyde at RT with or without AR was efficient for 12 antibodies/proteins out of 13, and considered as the best protocol for 10 out of them. Fixation with 0.5% formaldehyde alone for 30 min was efficient for 10 antibodies and considered as the best protocol for five antibodies. Two AR methods combined with 0.5% formaldehyde improved detection for four antibodies: 0.5% SDS in water at RT for 5 min and 0.1% trypsin in PBS at 37 °C for 5 min. Fixation with methanol alone was efficient for six antibodies, and considered as the best protocol for two antibodies.

### Cell cycle protein expression

#### Proliferation markers

**Figure 3:**
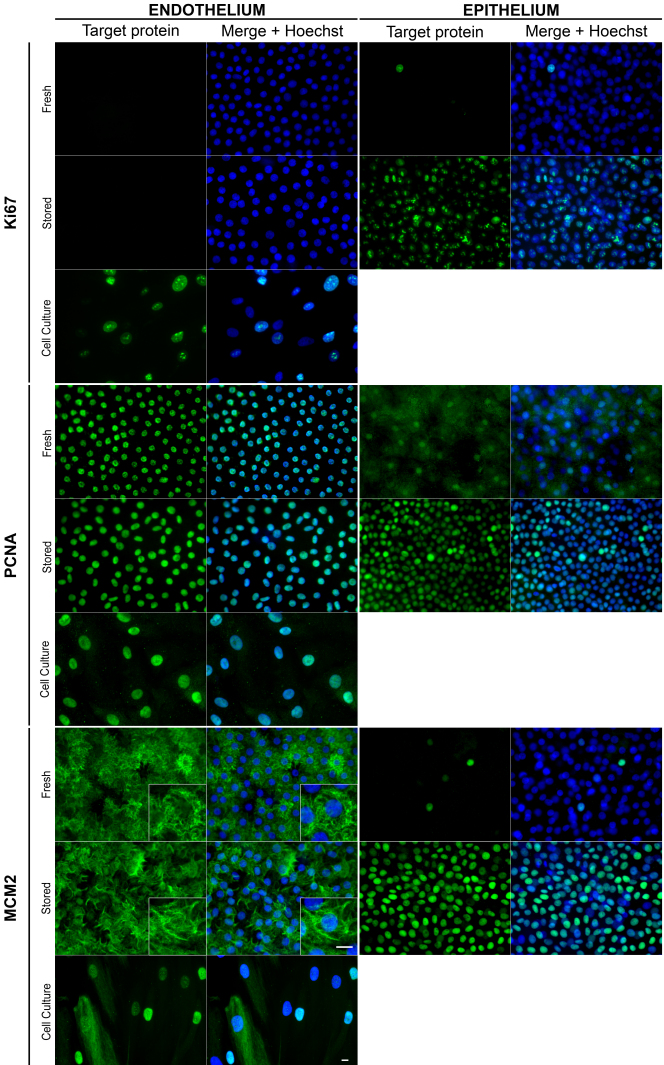
Optimized immunolocalization of proliferation markers in endothelial cells on flat-mounted whole human corneas. Only the best combination of fixative and epitope retrieval is shown for each antibody (see Table 4). Original magnification 40× except insets (96×). Bar 10 μm.

We were first interested in analyzing the presence and localization of proliferation markers widely used in clinical histological studies, and selected Ki67, PCNA and MCM2 (Figure 3 and Table 5). Ki67 positive cells were not detected in ECs of whole corneas. A few positive cells were observed in the epithelium of fresh corneas with death-to-procurement time under 24 h. The number of Ki67 positive cells always increased dramatically in the epithelium of stored corneas. In in vitro ECs, Ki67 positive cells were numerous. Staining pattern characteristics of Ki67 were clearly visible in nuclei. Occasionally epithelial mitosis were observed, confirming completion of the cell cycle.

**Table 5:** Subcellular localization of the proteins involved in the cell cycle in endothelial cells and in both controls (in vitro endothelial cells and epithelial cells of whole flat-mounted corneas).

** **	**Endothelial cells**			**Epithelial cells**	
**Target protein **	**Fresh**	**Stored**	**In vitro cultures**	**Fresh**	**Stored**
Ki67	A	A	N	N	N
PCNA	N	N	N, N+C	N, C, N+C	N
MCM2	C	C	N, N+C	N	N
cyclin D1	C	C	N, N+C	C, N+C	N
cyclin E	N	N	C	N, C, N+C	N, C, N+C
cyclin A	C	C	N, N+C	C	N, C, N+C
p16	N	N	N, C, N+C	N, C, N/C	C
p21	A	A	N	N, C	N
p27	N	N	N, C, N+C	N, C, N+C	N, C, N+C

A: absence of detection; N: nuclear; C: cytoplasmic, N+C: both nuclear and cytoplasmic.

PCNA was essentially detected with an invariable signal in the nucleus of all ECs on both fresh and stored corneas and of in vitro ECs. Both nuclear and weak cytoplasmic expression was found in a few epithelial cells of fresh corneas. After storage, PCNA was detected in the nucleus of all epithelial cells.

The staining pattern of MCM2 differed totally in ECs and epithelial cells. It was mainly cytoplasmic in most of ECs of both fresh and stored corneas. It filled the lateral expansions of cytoplasm located at the basal Descemetic pole of ECs, giving a characteristic hairy aspect. MCM2 was preferentially detected in the nucleus of in vitro ECs, but cytoplasmic staining was also observed. In the epithelium, MCM2 was exclusively found in the nucleus of only a few cells on fresh corneas but in those of almost all cells on stored corneas.

#### G_1_ and S phase markers

**Figure 4:**
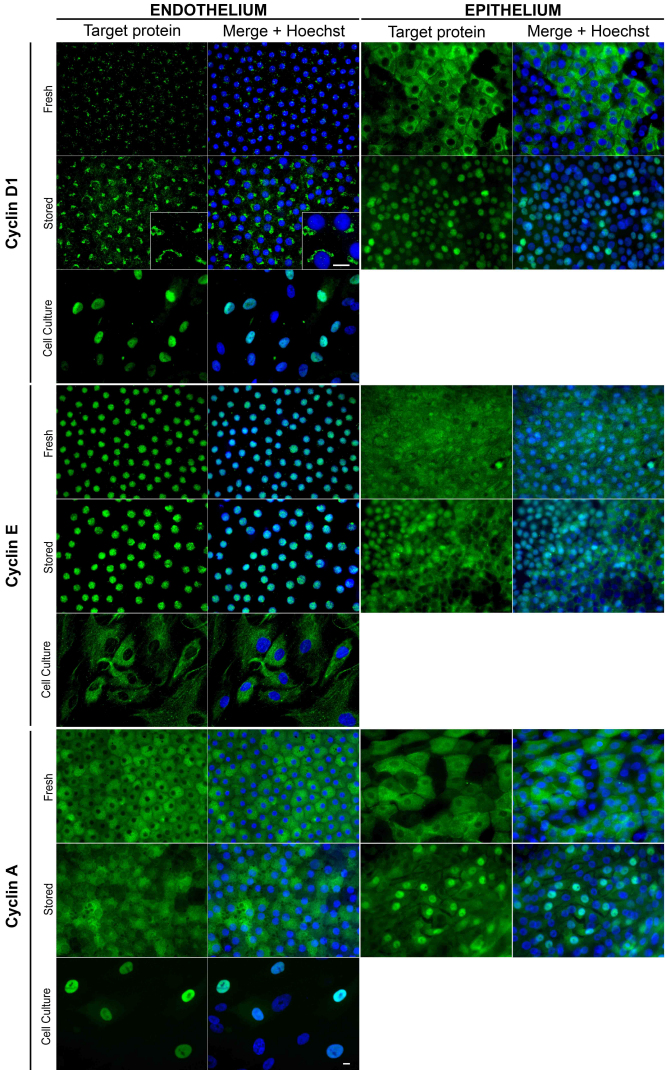
Optimized immunolocalization of cyclins D1, E, and A in endothelial cells on flat-mounted whole human corneas. Only the best combination of fixative and epitope retrieval is shown for each antibody (see Table 4). Original magnification 40× except insets (96×). Bar 10 μm.

We next analyzed the presence and localization of cyclins involved in the G_1_/S transition and S-phase, cyclin D1, cyclin E and cyclin A (Figure 4 and Table 5). The expression pattern of both cyclin D1 and cyclin E also differed totally in ECs and epithelial cells. Surprisingly, with the monoclonal rabbit antibody (ab16663; Abcam) no staining was observed in ECs whatever the sample preparation. With the polyclonal rabbit antibody (ab31450; Abcam) cyclin D1 was absent or had dim paranuclear staining in fresh corneas’ ECs, which increased, after storage. Nuclear and weak cytoplasmic staining was found in in vitro ECs. In epithelium, with both antibodies, cytoplasmic localization was observed on fresh corneas. After storage, cyclin D1 had a predominant nuclear localization in almost all epithelial cells.

Cyclin E was homogeneously detected in the nucleus of all ECs on both fresh and OC corneas but exclusively in the cytoplasm of in vitro ECs. In contrast, in the epithelium it was heterogeneously detected either in the cytoplasm, the nucleus or both and OC seemed to increase nuclear staining.

Cyclin A was found in almost all ECs on both fresh and stored corneas with a cytoplasmic localization. Hexagonal cytoplasmic patterns were observed in ECs on fresh corneas. Nuclear staining was predominant in in vitro ECs. In the epithelium, cyclin A was mostly cytoplasmic on fresh corneas, but accumulated in the nucleus in numerous cells after storage.

#### Cell cycle inhibitors

**Figure 5:**
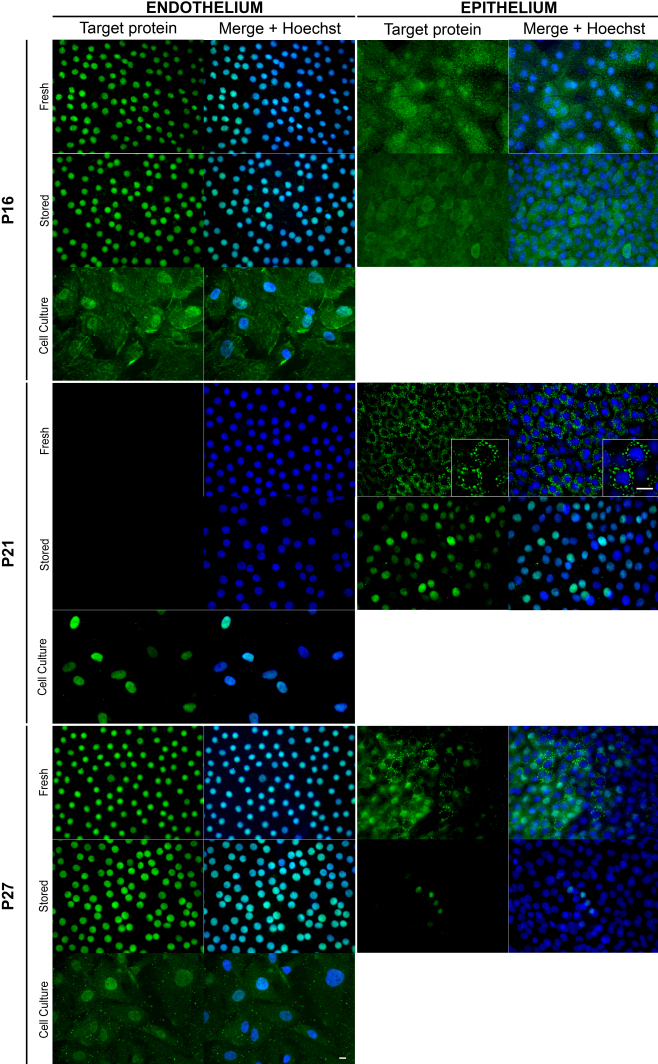
Optimized immunolocalization of three cyclin dependent kinase inhibitors p16, p21, and p27 in endothelial cells on flat-mounted whole human corneas. Only the best combination of fixative and epitope retrieval is shown for each antibody (see Table 4). Original magnification 40× except insets (96×). Bar 10 μm.

We next analyzed the presence and localization of cyclin-dependent kinase inhibitors, p16, p21, and p27 (Figure 5 and Table 5).

p16, a Cdk4 and Cdk6 inhibitor that play a role at the G_1_/S transistion was detected homogeneously in the nucleus of all ECs of both fresh and stored corneas. Weak cytoplasmic or both nuclear and cytoplasmic staining was observed in in vitro ECs as well as in epithelial cells on fresh corneas. After storage, the nuclear accumulation of p16 disappeared in all epithelial cells.

We found no p21, a Cdk1, and Cdk2 inhibitor, in ECs of fresh or stored corneas, with any of the three antibodies assessed under the various sample preparations. Nevertheless, p21 was found in the nucleus of in vitro ECs in an identical manner with all three antibodies, but the best results were obtained with the mouse monoclonal antibody (#2946; Cell Signaling). In epithelial cells, p21 was predominantly found in the cytoplasm of fresh corneas, with a particular granular pattern in the most superficial cells (especially with death-to-procurement time below 24 h). After storage, p21 was detected exclusively in the nucleus of epithelial cells.

p27, also a Cdk1 and Cdk2 inhibitor, was homogeneously detected in the nucleus of all ECs of both fresh and stored corneas. It was detected in both the nucleus and cytoplasm of in vitro ECs. In epithelial cells on fresh corneas, p27 was heterogeneously detected in cytoplasm, nuclei or both. Similarly to p21, cytoplasmic localization had a particular granular pattern in epithelial cells on fresh corneas with a short death-to-procurement time. After storage, p27 staining dramatically decreased and concerned only a few nuclei.

## Discussion

Through systematic testing, on a very large series of human corneas, of most of the fixatives and AR methods that we felt were applicable to the cornea, we determined the optimal preparations for several cell cycle control proteins.

### Optimization of immunolocalization on flat-mounted whole corneas

Endothelial immunostaining on flat-mounted corneas offers many advantages over that done on conventional histological cross-sections, but requires adaptation of the usual protocols due to the specificities of the endothelium. IL on flat-mounted whole corneas (intact cell monolayer on intact tissue) possesses the characteristics of both IHC (tissue) and ICC (intact cells). Contrary to IHC, where the content of cross-sectioned cells is easily visible, membrane permeabilization is required for intact ECs of whole flat-mounted corneas to diffuse bulky antibodies and secondary labels. This may be achieved by certain fixatives alone (altering lipids) or requires an additional step of membrane permeabilization. Moreover, while dangers of over- [11] and underfixation [22] have been highlighted for IHC on formalin-fixed tissue, they have not been discussed for a superficial cell layer such as ECs. These form a monolayer of cells that are strongly joined together but adhere relatively poorly to their Descemet membrane. They withstand certain reagents better than cells cultured in vitro on slides, but less well than cells nested in tissue mass. To preserve the integrity of this fragile monolayer, we found a compromise between tissue fixation, permeabilisation of cell membranes to give access to antibodies, and restricted use of AR techniques, which, though useful, carry a high risk of cell detachment or tissue retraction, especially those involving high temperature. In particular, we showed that the most common fixation method, 4% formaldehyde, did not achieve correct localization of most of the proteins studied, irrespective of their cell compartment, probably because there is high and rapid cross-linking of antigenic targets in the directly acccesible superficial cells [23].

We did not find a universal protocol able to satisfactorily reveal all antigens in ECs. Nevertheless, we selected several efficient solutions that could help standardize immunostaining of ECs and facilitate inter-laboratory comparisons. Notably, 0.5% formaldehyde with or without AR or methanol at RT for 30 min are the three simplest and most efficient protocols, as all antigens tested seemed correctly localized by one or another of the protocols. The efficacy of a low concentration of formaldehyde had been described for immunostaining on cytologic preparations [18]. It seems therefore that overfixing was the main obstacle during IL on ECs of flat-mounted corneas.

Interestingly, AR with SDS greatly improved immunolocalization of the three nuclear proteins H3, PCNA, and P16. SDS is a detergent, used in the SDS–PAGE technique and DNA extraction, because it can disrupt non-covalent bonds between proteins, and between proteins and DNA, a property also useful for AR. Histone H3 is one of the proteic constituents of chromatin, while PCNA can be presented in chromatin-associated form [24], and P16 can directly interact with PCNA in nuclei [25]. Our data suggest that SDS enhanced staining quality after formaldehyde fixation by unmasking the epitopic fragments of histone H3 or PCNA, which are probably hidden by their physical interaction with DNA.

We used two complementary positive controls for primary antibodies [20]: epithelial cells of the same cornea, observed at the same time on the opposite side of the flat-mount, and human corneal EC in primary cultures. Both were useful to distinguish negative staining of EC due to absence of expression or due to an unadapted protocol. Observation of the epithelial layer was particularly useful because it was submitted to exactly the same preparation as EC, as corneal pieces were entirely immersed in the different solutions. The difficulty relating to the multilayered organization of the corneal epithelium was taken into account during interpretation. Use of a non-confocal microscope was in this respect an advantage, as we could observe two or three layers of cells at the same time. In vitro ECs did not behave in the same way: stronger fixation (4% formalhedyde) was necessary to avoid cell detachment during the rest of the process. Why 4% formaldehyde allows perfect localization in in vitro ECs and not in situ, where a lesser concentration (0.5%) is far more appropriate, remains unclear. The surface coating layer present in vivo on top of the EC plasma membrane, comprising, among other molecules, membrane proteins, proteoglycans and glycoproteins [26] may form after 4% formaldehyde fixation, a layer relatively impermeable to most of the antibodies.

### Expression of the ten cell cycle proteins

To the best of our knowledge, investigation of a large range of proteins involved in the cell cycle had never been performed in fresh human corneas fixed immediately after procurement in donors, without being stored before study. Only Ki67 (clone SP6; Labvision, Fremount, CA) and PCNA (clone PC 10; Dako, Glostrup, Denmark) had been studied on 4% PFA-fixed cross-sections of fresh corneas, but with extended postmortem time (29 to 163 h) [27] and only PCNA on cross-sections of fresh corneas (postmortem time not available) [28]. Modifications in protein expression profile induced by any storage medium (4 °C or OC), even when storage is short, cannot be ruled out. By avoiding this storage time, one is sure to stay as close as possible to physiologic conditions, except for the postmortem metabolic changes that inevitably also occur. Only the study of corneas retrieved from enucleated globes for posterior tumor would ensure true physiologic conditions, but these cases are fortunately scarce and do not provide a sufficient source of human tissue.

We compared fresh corneas with similar corneas after several weeks of storage in OC and highlighted several differences never described before. Conventional OC media are derived from Dulbecco’s modified Eagles minimum essential medium supplemented with 2% FCS necessary to ensure EC survival up to 5 weeks. They also stimulate wound healing by cell migration and enlargement [29] but are not known to stimulate EC proliferation. Only in a very specific OC medium containing 8% FCS has EC proliferation been described in situ [27,28]. On the contrary, non-confluent in vitro cultures of human adult ECs contain numerous proliferating cells, explaining a profile totally different from EC in situ. Overall, the profile of cultured ECs is very similar to that of epithelial cells after OC, both being used as primary antibody controls [20]. During OC, the epithelial layer is highly activated and may completely regenerate even after long death-to-retrieval time [21]. Nevertheless, although cells proliferate, the number of cell layers gradually decreased during OC [30], explaining why we observed mainly basal cells where higher proliferative activity occurs. In this article, we have described both a method for systematically assessing antibodies on whole mount en face cornea, and staining patterns for a set of antibodies directed against cell-cycle proteins. We are well aware that the use of a single technique does not allow for definitive interpretation of data, and that additional primary antibody controls should be added. Several methods could be applicable to ECs, such as immunostaining after knocking down the specific protein expression with siRNA, a combination of immunostaining and fluorescent in situ hybridization, or immunoblots, and will be the subjects for future studies, as they require substantial technical developments. The following interpretation of our data should therefore be seen as open avenues for further investigations.

The Ki67 antibody was originally identified as a reliable marker for a nuclear antigen present in proliferating cells, from late G1 to M phase, and is therefore approved as a ubiquitous proliferation marker [31,32]. As expected, no Ki67 staining was observed in ECs of fresh and stored corneas, whereas it was abundant in epithelial cells and in vitro ECs, two situations where cells proliferate. Ki67 positive ECs and even mitosis have previously been described on wholemounts of human corneas, with clone MIB-1 by Zymed (San Francisco, CA), in experiments of wound healing in presence of 10% FBS and growth factors [33]. In this study, corneal pieces were fixed for 10 min in ice-cold methanol and incubated in 1% Triton X-100 to permeabilize the cells. We also found methanol to be a possible fixative for Ki67, but 0.5% formaldehyde without subsequent cell permeabilization seemed better. From the Ki67 pattern, it appears that whereas the epithelium is highly proliferative in OC, the endothelium is devoid of proliferating cells either in fresh or in stored corneas.

PCNA is another proliferation marker in a wide variety of cell types whose accumulation in the nucleus begins early in the G1 phase. It is a cofactor of DNA polymerase delta [34] but also has numerous other functions in response to DNA damage, cell cycle control, chromatin assembly and cohesion, and in other functions [24]. In ECs, PCNA had only been studied on cross-sections [27,28]: PCNA positive ECs were found in long-term OC corneas but not in fresh corneas [28]. On the contrary, we observed homogeneous nuclear PCNA staining in all ECs of fresh and stored corneas, indicating that PCNA may not be considered as a proliferation marker in this population, whereas staining was more typical in epithelial cells with an increase of nuclear staining after OC. The role of PCNA in ECs remains however to be determined but could be related to nuclear oxidative DNA damage that accumulates in ECs throughout their life [35]. Furthermore, there could be a relationship with the ubiquitous nuclear localization of p16 that we found in ECs of fresh and stored corneas, as p16 can interact directly with PCNA and inhibit DNA polymerase delta activity [25].

MCM2–7 are highly conserved proteins, which form a hexameric complex with replicative helicase activity [36] involved in the regulation of DNA duplication [37]. MCM2 is undetectable in G_0_, and accumulates in the nucleus during G_1_, peaking on S-phase entry [38], which explains its role as a proliferation marker. MCM2 is then eliminated from the nucleus during the S phase. In ECs in situ, we found no nuclear MCM2 but dense fibrillary cytoplasmic staining, unmodified after storage. The irregular shape of ECs was due simply to the filling by fluorescent markers of the irregular basolateral cytoplasmic expansions previously described [39,40]. In contrast, MCM2 was always detected exclusively in nuclei of the proliferative epithelial cells and preferentially in nuclei of in vitro ECs. Incidentally, a similar pattern in cultured ECs had already been described [41]. Interestingly, the sequestration of MCM proteins in cytoplasm and therefore the inability of MCM to initiate DNA synthesis is one of the mechanisms by which progesterone inhibits estrogen-induced uterine epithelial cell proliferation [42]. We propose that MCM2 sequestration in ECs could play a role in G_1_-phase arrest. Nuclear localization of MCM2 had previously been described in nuclei of ECs using corneal pieces fixed for 10 min in ice-cold methanol and incubated in 1% Triton X-100 to permeabilize the cells (MCM2 antibody; BD PharMingen, San Diego, CA) [7]. In the latter experiments, designed to analyze proliferation in response to a calibrated wound, corneas initially stored at 4 °C for several days were subsequently incubated at 37 °C in culture medium supplemented with 8% FCS and various growth factors. MCM2 positive nuclei were observed between 36 to 72 h after wounding and only in ECs close to the wound. These results do not contradict our own, since our OC conditions differ greatly from these proliferation-promoting conditions. In our hands, only 0.5% formaldehyde was able to make the Abcam MCM2 antibody react. Methanol did not help reveal cytoplasmic MCM2.

Cyclins and their catalytic partner cyclin-dependent kinases (CDKs), regulate cell cycle progression in eukaryotes. The three D cyclins (D1, D2, D3) accumulate during G_1_ phase in response to mitogenic stimulation, and assemble with CDK4 and 6. Cyclin D1 is an important actor in G_1_ to S progression by inactivating the cell cycle-inhibiting functions of the retinoblastoma protein through phosphorylation. In addition, cyclin D/CDK complexes sequester CDK inhibitors such as p21 and p27. Nevertheless, once assembled with CDK within the cytoplasm, complexes need to be imported into the nucleus to be active. For example, it has been demonstrated that the prevention of their nuclear import plays a critical role in the maintenance of cardiomyocytes in their non-proliferative state acquired soon after birth [43]. It has also been shown that postmitotic normal neurons lose their ability to import cyclin D/CDK complexes into the nucleus and that this ectopic cytoplasmic localization plays a role in neuron survival [44]. Aside from its role in cell cycle control, cyclin D1 also possesses other CDK-independent functions [45] such as chromatin remodelling, cell metabolism, and cell migration [46]. The exclusive cytoplasmic localization of cyclin D1 that we found in all ECs, without nuclear translocation after storage whereas it localized in nuclei of proliferating in vitro ECs and in basal epithelial cells after storage, agrees with its non-proliferative status but suggests that cyclin D1 has CDK-independent functions in ECs. It also suggests that ECs might have passed the restriction point (after which, in a normal cell cycle, cyclin D1 is exported from the nucleus).

Cyclin E/CDK2 complexes act normally after cyclin D/CDK4–6 complexes during G_1_ phase. Their activity rises in late G_1_ phase, peaks at G_1_/S transition, and declines quickly during the S phase [47,48]. Nevertheless, cyclin E accumulation in nuclei with inactive cyclinE/cdk2 complexes is compatible with arrest in the G_1_ phase related to senescence [49] and when overexpressed in nontransformed HC11 mouse mammary epithelial cell line, cyclin E localizes exclusively in nuclei, induces an increase in p27 expression, and inhibits cell proliferation [50]. Our results, showing constant localization of cyclin E in nuclei of all ECs in fresh and stored corneas, are therefore compatible with non-proliferative status, and suggest again that ECs in situ might be arrested after the restriction point and before the entry in S-phase. In epithelial cells, staining was heterogeneous and nucleocytoplasmic in most cells in fresh corneas with a predominant nuclear translocation after storage, suggesting progression through the cell cycle. In in vitro ECs cyclin E was mostly localized in the cytoplasm, suggesting that they crossed the restriction point of the cell cycle.

Cyclin A is nomally expressed from late G_1_ to late G_2_ phases. Cyclin A/CDK complexes have substrates in both the nucleus and the cytoplasm and shuttle between the two compartments [51]. It initially assembles with CDK2 to form a complex imported in the nucleus, where it phosphorylates nuclear substrates implicated in DNA replication, thus promoting progression through the S phase [52]. In proliferating epithelial cells and in vitro ECs, cyclin A is mainly nuclear which is a hallmark of proliferating cells. We found it exclusively expressed in the cytoplasm of ECs of fresh and stored corneas, which again hints at cells having passed the restriction point. It is possible that the cytoplasmic retention of cyclin A hinders progression through S-phase. It would be interesting to assess cyclin A/CDK complexes activity in ECs in situ, as cytoplasmic substrates of the complexes might play a role in ECs physiology.

Our results for cyclins D1, E and A in whole corneas were consistent with the previous studies by Joyce et al. on cross-sections of corneas from seven donors aged 6 weeks to 67 years and stored at 4 °C during 24 to 36 h (Antibodies from Upstate Biotechnology, Lake Placid, NY) [53,54]. Flat mounts now allow clearer subcellular localization as well as observation of a larger population of ECs.

We detected p16 in the nuclei of ECs of both fresh and stored corneas. This is consistent with previous results of IHC done on cross-sections of human corneas, although subcellular localization was in either cytoplasm [54] or nuclei [55,56] of ECs. P16 inhibits cyclin D/CDK4–6. It is thus considered to have a pivotal role in G_1_-phase arrest of ECs and stress-induced premature senescence [57]. Furthermore, p16 could interact directly with nuclear PCNA found in ECs and inhibit DNA polymerase delta activity [25]. In epithelial cells, cytoplasmic translocation occurred after OC, corresponding to prominent proliferative status. In in vitro ECs, p16 was found in both nuclei and cytoplasm, indicating cytoplasmic translocation compatible with cell cycle progression.

We did not detect p21 in ECs of flat-mounted fresh or OC-stored corneas despite systematic assessment of a wide variety of fixatives and AR and the use of three antibodies. P21 positivity in the nuclei of epithelial cells of the same corneal piece and in in vitro ECs eliminates a false-negative result in ECs. This apparently contradicts two previous studies that used IHC on cross-sections of 4 °C stored human corneas. In one case, a p21 antibody C-19 from Santa Cruz Biotechnology was used on six corneas stored at 4 °C and confirmed by western blots of proteins extracted from in vitro EC cultures [55]. In the second case, a p21 mouse monoclonal antibody from Lab Vision Corp (Fremont, CA) was used on four corneoscleral rims left off after corneal graft and confirmed with quantitative RT–PCR on RNA extracted from other corneo-scleral rims [56]. Both found nuclear staining for p21. It remains unclear why we did not reveal p21 in ECs in flat-mounted cornea methods. It is possible that fresh and OC corneas with preserved metabolic activities may behave differently from corneas stored for several days at 4 °C. As we found that in vitro EC cultures expressed p21 in nuclei, it is not surprising that WB was found positive by Enomoto et al. [55]. Moreover, the presence of p21 transcripts in ECs from whole corneas is not incompatible with post-transcriptional alterations resulting in absence of protein expression. At present, no other study has revealed p21 expression using immunolocalization in ECs. A direct comparison of paired corneas, one stored at 4 °C and the other either studied fresh or after OC, seems necessary to answer the question. We detected p21 in the nucleus of proliferative cells: epithelial cells of OC corneas and in vitro EC cultures. P21 is one of the main CKIs implicated in late G_1_-phase arrest by inhibiting cyclin E/CDK2 complexes [58], but p21 can also contribute to initial G1 progression by promoting the nuclear import of cyclin D/CDK4–6 complexes [59] and by preventing the nuclear export of cyclin D [60].

P27, another CIP/KIP family member, also strongly inhibits cyclin E/CDK2 complexes and arrests the cell cycle in G_1_ [61]. It is implicated in cell cycle arrest triggered by contact inhibition and TGFβ [62]. Its role has been demonstrated in rats [63] and the protein has been detected in nuclei of ECs on cross-sections of two corneas from donors aged 18 and 74 years [55] and in one cornea from a 54-year-old donor [56]. The three corneas had been stored shortly at 4 °C beforehand. In our study, in epithelial cells, the near-complete disappearance of p27 after OC strongly suggests cell cycle progression as, at the end of the G_1_ phase, cyclin E/CDK2 complexes (which we found in epithelial cell nuclei and cytoplasm) phosphorylate p27 that is subsequently ubiquitinated by Skp1/Skp2 and degraded by the proteasome [64]. In cultured ECs, p27 in both nuclei and cytoplasm also suggests possible progression beyond the restriction point.

In conclusion, IL on flat-mounted whole corneas seems possible for a large number of proteins, provided that substantial adaptations in corneal fixation and further processing are adopted. Continued efforts to refine methods of antigen preservation or retrieval in human corneas will ultimately allow standardization of IL and elimination of inter-laboratory discrepancies in results, thus allowing accurate research. 0.5% formaldehyde or methanol without AR should be recommended as first-line fixatives during experimental developments, given that in situ ECs seem particularly sensitive to overfixation by aldehydes and that AR methods often harm the integrity of the endothelial monolayer. Conventional OC medium containing 2% FCS does not modify the non-proliferative status of ECs, which is compatible with G_1_-phase arrest. Nevertheless, our data suggest that ECs are arrested after the restriction point in the G_1_ phase. The role of MCM2 in cytoplasm, the subcellular localization of cycle D1, and the absence of p21 are the main characteristics highlighted by our optimized protocols. Further studies are necessary to explain these particularities of fresh and OC-stored corneas.
